# Formaldehyde Hemiacetal Sampling, Recovery, and Quantification from Electronic Cigarette Aerosols

**DOI:** 10.1038/s41598-017-11499-0

**Published:** 2017-09-08

**Authors:** James C. Salamanca, Ian Munhenzva, Jorge O. Escobedo, R. Paul Jensen, Angela Shaw, Robert Campbell, Wentai Luo, David H. Peyton, Robert M. Strongin

**Affiliations:** Department of Chemistry, Portland State University, 1719 SW 10th Ave., Portland, OR 97201 USA

## Abstract

The electronic cigarette solvents propylene glycol and glycerol are known to produce toxic byproducts such as formaldehyde, acetaldehyde and acrolein. However, the aerosol toxin yield depends upon a variety of chemical and physical variables. The formaldehyde hemiacetals derived from these solvents were reported as major electronic cigarette aerosol components by us in 2015. In the study described herein, the formaldehyde hemiacetals were found at higher levels than those of free formaldehyde via an orthogonal sample collection protocol. In addition, the common aldehyde collection methods for electronic cigarettes, such as impingers and sorbent tubes containing DNPH, significantly underestimate the levels of formaldehyde. The reason for this is that formaldehyde hemiacetals follow other reaction pathways, such as the formation of a less reactive full cyclic acetal catalyzed by the acidity of the DNPH solution and the silica. We found that formaldehyde hemiacetals are a considerable fraction of the total formaldehyde produced in electronic cigarette that cannot be determined accurately by DNPH derivatization methods. Although the health effects of the hemiacetals are not yet known, they warrant further investigation.

## Introduction

Tobacco smoking is the leading cause of preventable death in the US^1^. Electronic cigarettes have been promoted as a healthier alternative to smoking, however little is known about their long-term health effects. A concerning current trend is the manufacture of customizable electronic cigarettes with increasingly lower resistance heating coils and higher operating temperatures that can enhance the likelihood of exposure to higher aerosol toxin levels^2, 3^. One means of understanding the toxicity of e-cigarettes while long-term epidemiological studies are underway is the elucidation of the chemical profiles of e-cigarette aerosols^2–14^. However, a major challenge is the lack of standardized analytical protocols. This issue has led to wide variations in interlaboratory results and has contributed to the dichotomy in the literature about electronic cigarettes and their potential health effects. For example, a comparison of five studies published the same year (2014) of formaldehyde (HCHO; and other carbonyl) analyses using DNPH sorbent tubes and/or DNPH impingers, showed that levels of detected HCHO ranged from a low of 3.2–3.9 ng/puff to a high of 660–3400 ng/puff^2^. Since e-cigarette aerosols consist of both gaseous and particulate matter (PM), one cause of the discrepancies in these results is that sorbent tubes were not designed for the analysis of PM. This should not affect gaseous HCHO analysis. However, HCHO can exist in equilibrium with several common adducts. Some of these, such as hemiacetals **1a**–**d** and methane diol (HCHO monohydrate), will partition into the PM phase of e-cigarette aerosols^15^. Moreover, unless each of the equilibrium forms of HCHO is converted to the carbonyl form, they cannot be detected using DNPH (both sorbent tube or impinger methods).

**Figure 1:**
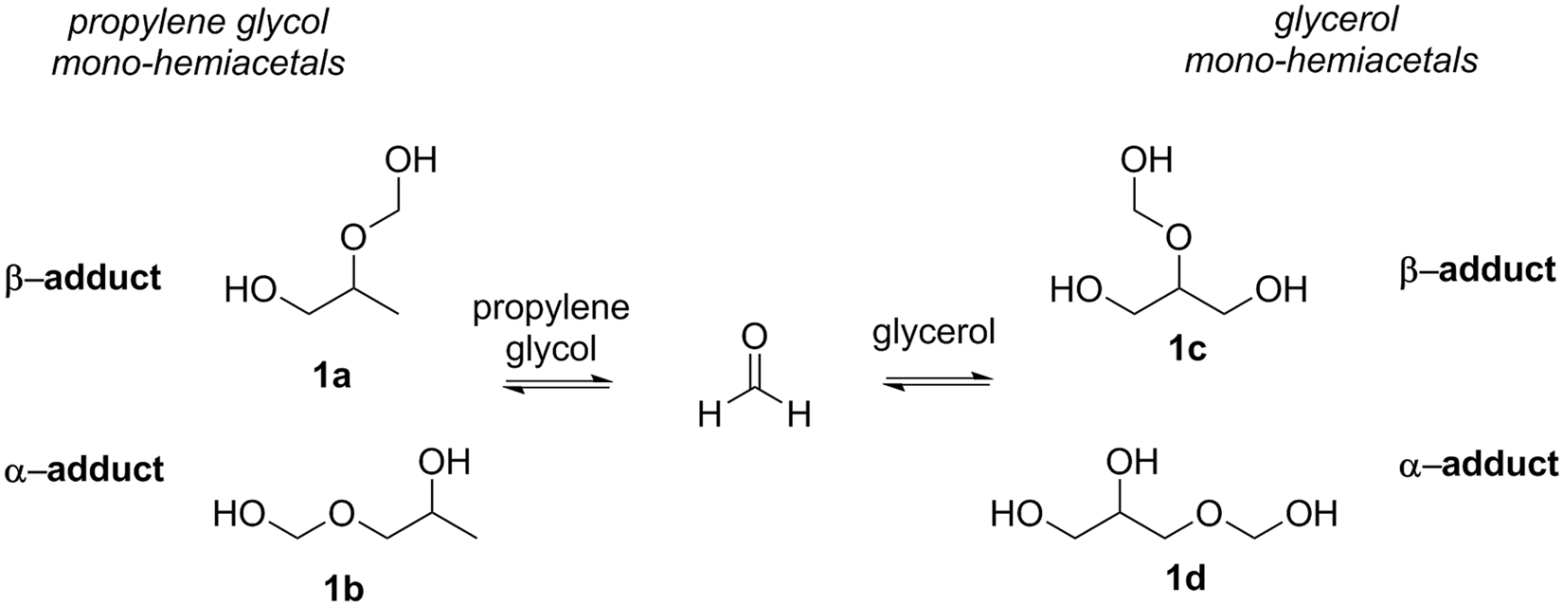
The formation of hemiacetal isomers **1b** and **1d** from PG and the isomers **1c** and **1d** from GLY. PG and GLY are the most common e-cigarette solvents. HCHO is well-known to be formed via the partial thermal degradation of PG and GLY during the vaping process. Hemiacetals are stable enough to be detected by NMR spectroscopy after aerosol generation, but are hydrolyzed to the component alcohols and HCHO when diluted in H_2_O.

We previously reported the formaldehyde hemiacetal isomers (**1a**–**d**, Fig. 1) in the aerosols derived from a variable voltage electronic cigarette above a threshold power setting^6^. The levels of **1a**–**d** were higher than those of gaseous HCHO found in traditional cigarettes. There was a response to the report via a published study that attempted to reassure e-cigarette users that there is relatively little danger of inhaling any more than “minute” levels of toxins, as long as they are unable to taste them^16^. However, the primary focus of the report was the finding of hemiacetals (**1a**–**d**) as major aerosol products. In a more recent investigation, we showed that **1a**–**d** can form under relatively benign, single puff conditions^8^. During the preparation of this manuscript, another significant report appeared showing that **1a**–**d** levels were indeed a potentially concerning proportion of the total HCHO (in the 22–45% range) found in e-cigarette aerosols, specifically when using newer devices possessing increasingly higher power output capabilities^3^. However, as is shown herein, the relative levels of **1a–d** to HCHO produced by e-cigarettes can be even higher, because they are dependent on many key variables such as sampling and analytical techniques. Monitoring the levels of **1a–d** is significant not only to account for various possible routes of HCHO exposure, but also because **1a–d** will be inhaled as aerosol droplets^15^ more deeply into the respiratory tract compared to gaseous HCHO^6^.

The hypothesis driving this investigation is that **1a–d** can be formed at higher levels than gaseous HCHO in e-cigarette aerosols, based on well-established fundamental chemistry of alcohols and HCHO. This has been studied in detail by Balashov and co-workers^17^, who demonstrated that formaldehyde hemiacetals are by far the major components in equilibria involving carbonyl HCHO and excess levels of alcohols, as is the case in e-cigarettes. During the course of this investigation, we employed a new sampling and analytical method that enables the collection and determination of HCHO and its hemiacetals within the same experiment. Major findings include the facts that e-cigarette aerosol analyses that are based solely on DNPH cartridges or DNPH impingers significantly underestimate levels of **1a**–**d** by *ca*. 35–45%, and that hemiacetals can be produced in e-cigarette aerosols at levels that are up to ~14 times higher than those of gas-phase carbonyl HCHO.

## Methods

### E-Cigarette Device and E-liquid Composition

The e-cigarette used in this study was comprised of a Vaporfi Vox TC battery unit and a KangerTech ProTank II “glassomizer” (also referred to as a glass clearomizer) with a 2.2 Ω resistance CC clear cartomizer coil units. Wattage settings of the battery unit used were 10 W and 15 W. These conditions were chosen to produce amounts total HCHO that could enable them to be conveniently distinguished, with the intent to produce a comparison between various sampling and analytical methods. E-liquid was composed of a 2 mL solution of a 1:1 mixture of food grade PG/GLY purchased from Sigma-Aldrich. The 50/50 ratio of PG/GLY is a benchmark median that has been used in our preceding studies^6, 8^ as well as in the related prior work of others^10^ employing a 50:50 PG/GLY commercial e-liquid.

### Smoking machine

An SCSM-STEP single cigarette-smoking machine (CH Technologies, Westwood, NJ) was used.

### DNPH Solution

DNPH solution was prepared in accordance with the CORESTA standardized method for DNPH stock solutions. DNPH was purified via recrystallization^18^. Approximately 1 g DNPH hydrate was weighed and added to 21.4 mL EtOH and warmed with magnetic stirring agitation. 28.57 mL EtOAc was added slowly with heat and stirring until all of the DNPH was dissolved. The warm solution was vacuum filtered and transferred to an Erlenmeyer flask and cooled overnight. Recrystallized DNPH was isolated using vacuum filtration. The crystals were placed in a desiccator to protect from moisture. Recrystallized DNPH (0.849 g) was added to 175 mL MeCN and 175 mL of H_2_O containing 3.5 mL phosphoric acid (85%). Fresh 250 mL DNPH stock solution was prepared weekly and stored in an amber flask at room temperature.

### Collection using DNPH sorbent tubes

The tubes used were DNPH-treated silica gel sorbent tubes containing bisectional 150/300 mg high-purity silica gel sorbent 6 mm × 110 mm (SKC Inc. Eighty Four, PA). A Phenex GF/CA 28 mm filter 0.45 µm (Phenomenex, Torrance, CA) was attached between the apparatus and the smoking machine. Two sorbent tubes were connected in series. 3 puffs were collected using a puff duration = 3.0 s, a puff interval = 30 s and a puff volume = 50 mL.

### Collection using impingers

Two impingers containing DNPH solution were connected in series and the puff collection performed the same way as with the sorbent tubes. The use of 2 impingers at room temperature is from the EPA, CORESTA methods for carbonyl quanitification. Using colder conditions DNPH crystallizes. Additional impingers were not necessary because we observed no products in a third impinger connected in series.

### Combined qNMR-DNPH collection and quantification

The electronic cigarette was connected to two cold-finger collection tubes in series at −78 °C (dry ice-acetone cooling mixture) followed by two impingers in series containing 20 mL each of DNPH solution. A Phenex GF/CA 28 mm filter 0.45 µm was attached between the apparatus and the smoking machine. 20 puffs were collected using a puff duration = 3.0 s, a puff interval = 180 s and a puff volume = 50 mL.

After every set of three puffs, there was a 3 minute interval to allow the aerosol to condense in the cold-trap. Particulate matter was extracted from the cold-trap collection tubes via rinsing with 1.2 mL DMSO-*d*_6_. The aerosol material from the cold-trap collection tubes was analyzed directly by ^1^H NMR. DNPH adducts in the DNPH impingers were collected and analyzed via HPLC. Each experiment was repeated 3 times at 10, and 15 W.

### HPLC analysis of DNPH impinger components

Before each run the syringe and the injection port loop were rinsed 3 times with MeOH and 3 times with MeCN. DNPH samples were analyzed and quantified using a Waters 1525 Binary HPLC Pump and a Waters 2996 Photodiode Array Detector. Analysis conditions: two SUPELCOSIL C-18, 25 cm × 4.6 mm, 5 µm particle size columns connected in series with a column heater at 40 °C. The mobile phase comprised of MeCN/H_2_O with a gradient system as follows: 0 min. 60/40; 7 min. 60/40; 25 min. 100/0, at a combined flow rate of 1 mL/min, with a 360 nm detection setting. The sample injection volume was 20 µL.

### qNMR spectroscopic analysis of cold trap components

^1^H NMR spectra were acquired using a 30° observation pulse, a relaxation delay of 60.0 s, and 64 scans, using a Bruker Avance II + 400 MHz or Avance III 600 MHz spectrometer. The main signal corresponding to the isomer mixture **1** resonating at 6.2 ppm (t) was integrated and compared to the internal standard 1,2,4,5-tetrachloro-3-nitrobenzene (TCNB) signal at 8.5 ppm (s). The purity (P), molar mass (M), number of nuclei (N) and weight (W) of the internal standard and **1** signals were calculated with their respective integral areas (I) in the equation^19^:

$${P}_{x}=\frac{{I}_{x}}{{I}_{std}}\cdot \frac{{N}_{std}}{{N}_{x}}\cdot \frac{{M}_{x}}{{M}_{std}}\cdot \frac{{W}_{std}}{{W}_{x}}\cdot {P}_{std}$$

### Synthesis of 1**a**–**d**

Compounds **1a**–**d** were synthesized to serve as analytical standards. They are in equilibrium with PG, GLY, H_2_O, HCHO, methanediol and each other. Their limited stability in solution at rt has been described previously^8^. The synthesis is derived from the procedure of Balashov^17^, but with either PG or GLY used as the reactant with HCHO instead of ethylene glycol. Conditions that Balashov used for mono-addition of HCHO were used. 20.5 mL PG or GLY and 1.15 mL H_2_O were added to a graduated cylinder. 2.10 g of paraformaldehyde was added to a round-bottom flask and attached to a serrated gas impinger submerged in the PG (or GLY): H_2_O mixture. Using a slow argon flow through the round bottom flask and impinging into the solution, paraformaldehyde was heated via a heat gun for 15 min, leaving a dark residue. The solution in the gradated cylinder was covered and stirred 24 h at rt. A 2.0 mL aliquot of the solution was diluted with 20 mL MeCN for HPLC purification as follows.

### Enrichment of 1a and 1b via HPLC

Crude samples of **1a** and **1b** were purified by HPLC using a Phenomenex Luna^®^ CN 250 mm × 10 mm HPLC-column (particle size 5 µm, pore size 100 Å) jacketed with a column heater at 40 °C. The mobile phase used was MeCN at a flow rate of 6.0 mL/min. The detection wavelength was set to 196 nm. The sample injection volume was 200 µL. A peak eluting at 2.78–2.94 min was collected over two purification cycles. After solvent removal, the product contained 59% of **1a** and **1b** (calculated from ^1^H NMR integration).

### Conversion of 1a–d to the DNPH-HCHO adduct via (a) DNPH sorbent tubes and (b) DNPH impinger solutions

(**a**) Sorbent tubes: Sorbent tubes were opened and the glass wool inside was removed. 27.0 μg of **1a** and **1b** were injected directly into the sorbent bed of the tubes. Residence times in the tubes were up to 2 h. The tubes were eluted with 5 mL MeCN. Solutions were filtered prior to HPLC analysis. (**b**) DNPH impinger solutions: In parallel experiments, 27.0 μg of enriched **1a** and **1b** was added to 5 mL of standard DNPH solution and filtered (after up to a 2 h sample residence time) for HPLC analysis.

In order to monitor the efficiency of the conversion of **1a** and **1b** to HCHO as the HCHO-DNPH adduct, 20 μL samples from the impinger or the sorbent tubes were analyzed and quantified by HPLC. The recovery of **1a** and **1b** was assessed in 100% PG or GLY (i.e., using the analytical standards). Other PG:GLY proportions do not affect recoveries in either the impingers or on the columns due to the very large excess of MeCN/H_2_O or MeCN present in each, respectively. Moreover, both the PG-hemiacetal and the GLY-hemiacetal have overlapping ^1^H NMR signals^8^.

## Results

### PM and gas collection from a vaping device

#### Collection using DNPH sorbent tubes

After collection, the sorbent tubes were eluted with 5 mL MeCN, the solution filtered and subjected to HPLC analysis. The amount of HCHO (µg/mg e-liquid) present was 4.15 at 10 W and 2.80 at 15 W.

#### Collection using impingers

After collection, the solution from each impinger was filtered and subjected to HPLC analysis. The amount of HCHO (µg/mg e-liquid) present was 8.83 at 10 W and 13.34 at 15 W.

#### A sampling method for simultaneous capture of DNPH-reactive and DNPH-unreactive aerosol components

**Figure 2:**
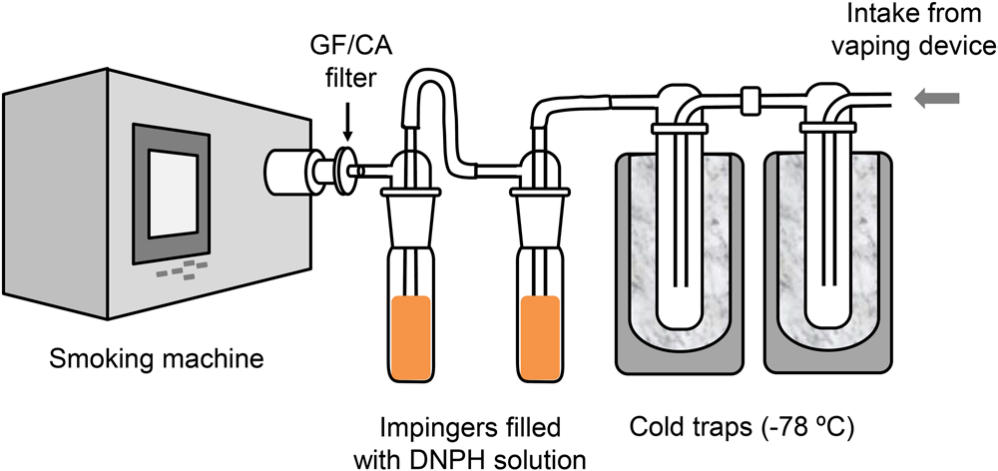
Sample collection apparatus for both the PM and gas phases.

In order to capture and analyze both DNPH-reactive and DNPH-unreactive HCHO and hemiacetal respectively an orthogonal trapping setup was employed. The e-cigarette aerosol was first exposed to a pair of cold traps (−78 °C) connected in series, followed by a series of two impingers containing standard DNPH solution. The second of the two impingers was attached to the smoking machine (Fig. 2). The cold traps enabled hemiacetals **1a**–**d** capture for direct analysis by ^1^H NMR after sample dilution with DMSO-*d*_6_. The DNPH impingers enabled DNPH-HCHO adduct formation and collection, followed by HPLC analysis.

**Figure 3:**
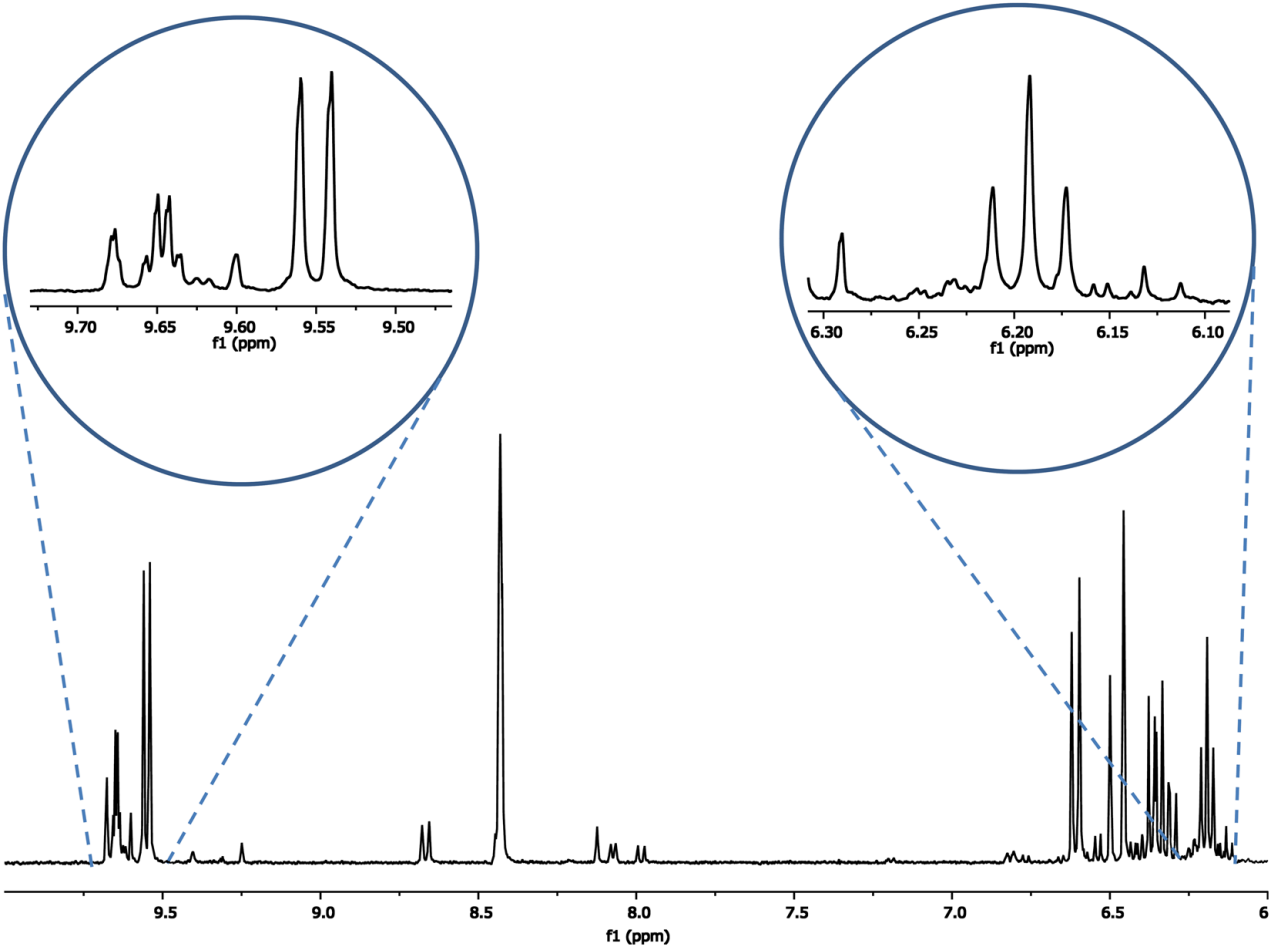
A representative ^1^H NMR spectrum of e-cigarette aerosol components captured in the −78 °C cold traps. No processing except for dilution with DMSO-*d*_6_ was performed on this sample. The inset on the right shows an expansion of the region containing the triplet peak at 6.19 ppm that corresponds to the major hemiacetal peaks. In the inset at left, an expansion of doublet centered at 9.55 ppm corresponds to acrolein. We have reported the ^1^H NMR spectra and chemical shifts of these and over a dozen aerosol components recently, noting that acrolein levels may be also be underestimated by DNPH methods, based on the relative prominence of its aldehyde proton resonance^8^.

**Figure 4:**
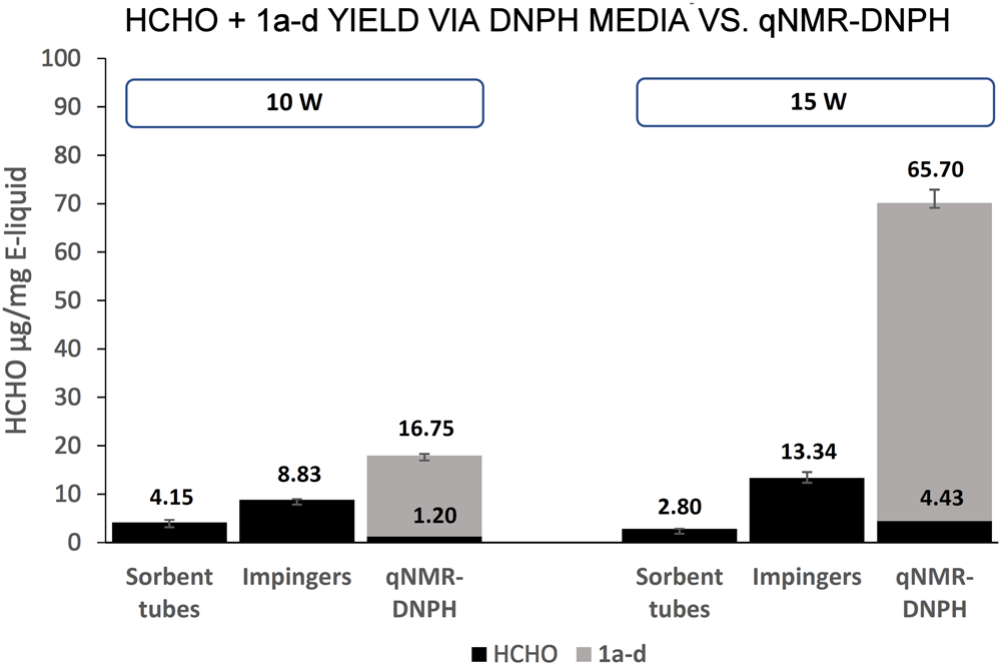
Comparison of total HCHO μg per mg e-liquid consumed in e-cigarette aerosol via DNPH derivatization methods (DNPH sorbent tubes or bubbling into impingers containing DNPH solution) versus a combined qNMR-DNPH derivatization method. The qNMR-DNPH hybrid method enabled the quantification of **1a–d** and HCHO in the same experiment. DNPH sorbent tubes produce the least amount of HCHO detectable, whereas the combined qNMR-DNPH method enabled the detection of a greater amount of HCHO than that of both DNPH solution and sorbent tubes. DNPH does not react with hemiacetals since they do not contain free carbonyls. The increase in yield at 15 W vs. 10 W is due to higher power: note that there is ~4x the HCHO detected at 15 W vs. at 10 W (1.20 µg at 10 W vs. 4.43 µg at 15 W) as well as ~4x (**1a**–**d** + HCHO) (16.75 µg at 10 W vs. 65.70 µg at 15 W). Both wattages have ~14x the proportion of **1a–d** to HCHO.

The amount of **1a**–**d** present in the e-cigarette aerosols, based on qNMR analysis of the cold trap fractions from triplicate experiments (Fig. 3), was 16.75 μg/mg e-liquid consumed at 10 W and 65.70 μg/mg e-liquid consumed at 15 W. The level of gaseous carbonyl HCHO, determined by HPLC analysis of the DNPH adduct, was 1.20 μg/mg e-liquid consumed at 10 watts and 4.43 μg/mg e-liquid consumed at 15 W. The ~14:1 ratio of excess hemiacetals **1a-b**: HCHO was thus consistent between the two wattages (Fig. 4). DNPH sorbent tubes enabled detection of the least amount of HCHO, whereas the combined qNMR-DNPH method enabled the detection of a greater amount of total HCHO (**1a**–**d + **carbonyl HCHO) compared to both DNPH impinger and sorbent tube methods.

#### Efficiency of the detection of 1a–d by DNPH impingers and SKC sorbent tubes

In order to determine how effectively the hemiacetals were converted to HCHO and detected by the DNPH methods, an analytical standard enriched (from PG) in **1a** and **1b** was applied to sorbent tubes and placed in DNPH solutions. The reaction with DNPH to form the HCHO adduct was rapid (<1 min), as evidenced by HPLC monitoring for up to 2 h. However, recovery of the hemiacetal standards from the impingers and the sorbent tubes was only 67% and 54%, respectively.

## Discussion

### Formaldehyde hemiacetal chemistry is unique

**Figure 5:**
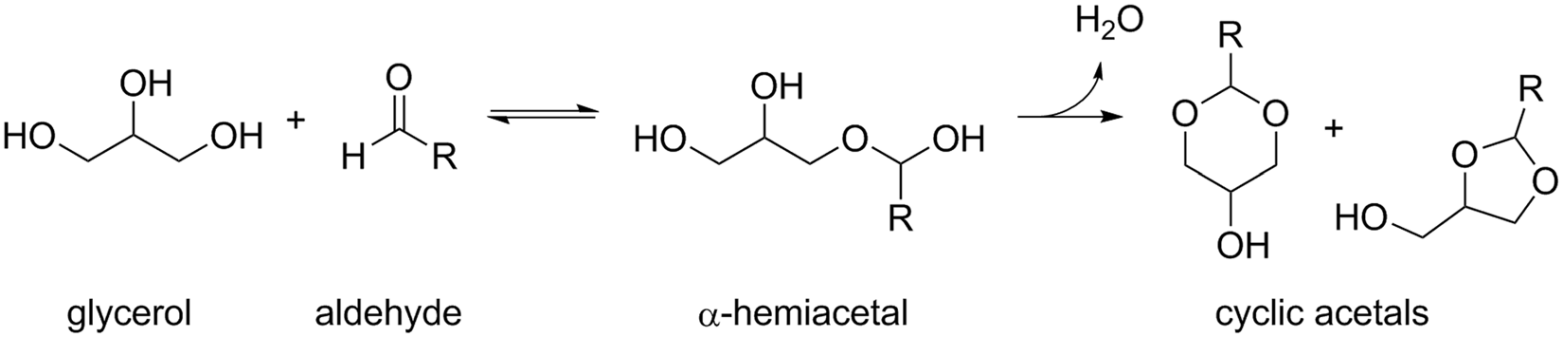
Hemiacetal and acetal formation, as described by Nef in 1904, between aldehydes that formed via GLY decomposition and GLY. Acetals that Nef identified included adducts of HCHO, acetaldehyde, and acrolein.

HCHO was identified as a decomposition product of electronic cigarette liquids in 2007, when Paine *et al*. described its evolution from the α-carbons of glycerol^20^. HCHO was identified as a degradation product of GLY by Nef in 1904^21^. Nef additionally observed the formation of *glycerinäthers*, cyclic acetals that formed via the reaction of various aldehyde decomposition products and GLY. The acetals formed via the reaction of aldehydes and polyols via hemiacetal intermediates (Fig. 5). Specific GLY acetals of HCHO and acetaldehyde had been observed prior to Nef’s study^22, 23^. In addition, the product of HCHO and PG is also well-known^24^. The mechanism of formation of HCHO, along with **1a**–**d** and 15 related products derived from PG and GLY oxidation and dehydration in e-cigarette aerosols was investigated and reported by us recently^8^.

**Figure 6:**
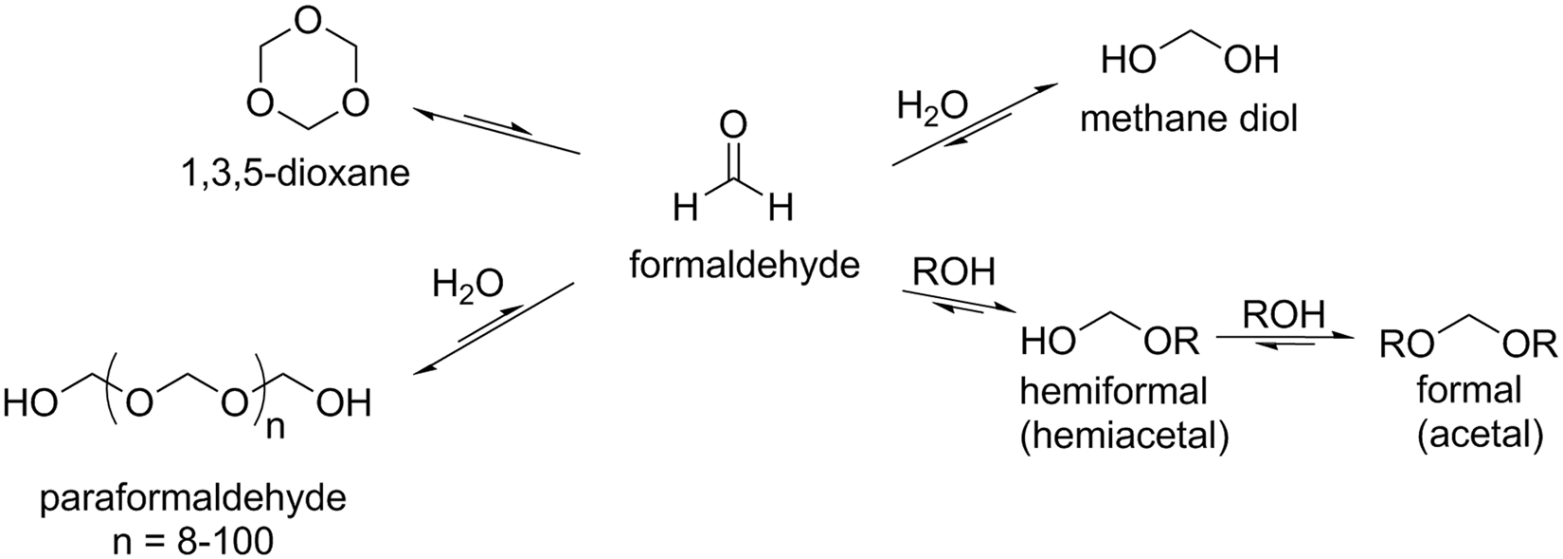
Formaldehyde and typical equilibria.

In addition to acetals and hemiacetals, HCHO reversibly forms a linear polymer, a cyclic trimer, and aqueous hydrate (Fig. 6) ^25^. Unless the reaction between DNPH and HCHO effectively promotes the other species present in the equilibrium to convert cleanly to HCHO via LeChatelier’s principle, the total level of an individual’s potential HCHO exposure will be underestimated.

### A higher yield of hemiacetals compared to carbonyl HCHO was observed in the e-cigarette aerosols, and is well-precedented in related HCHO reactions

**Figure 7:**
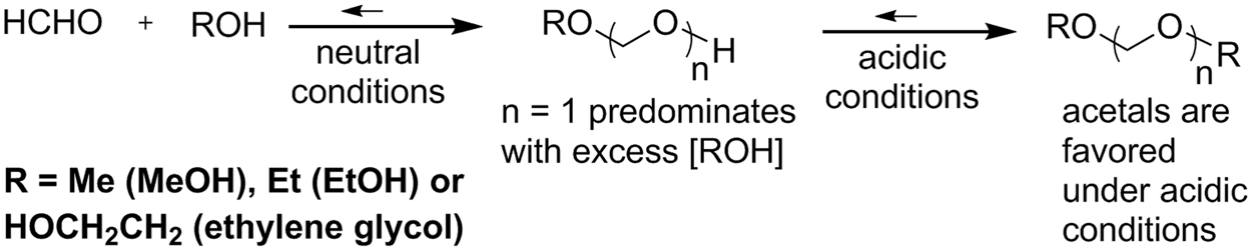
A prior investigation by Balashov determined that under neutral conditions hemiacetals are the predominant product in equilibria that involve HCHO and alcohols. Under acidic conditions, conversion of the hemiacetals to acetals is favorable. Acetals do not afford any detectable products after subjection to DNPH impinger and sorbent tube conditions, and thus embody a likely source of undetected **1a**–**d** and HCHO.

The yield of hemiacetals in aerosols was *ca*. 14 times higher than that of carbonyl HCHO at each of the 10 and 15 watt e-cigarette settings using our combined method. It is important to mention that there are inherent sensitivity differences for DNPH and qNMR analysis and the sampling size must be adjusted. In 2000, Balashov^17^ reported a detailed study of the equilibrium characteristics of neutral HCHO-H_2_O-alcohol (MeOH, EtOH and ethylene glycol) systems. He found that, irrespective of the proportion of HCHO: alcohol, HCHO was “almost completely bound into hemiacetals”. He stated that the hemiacetals (Fig. 7) converted to acetals in acidic media. Increasing the H_2_O concentration only led to an increase in the methane diol (Fig. 6) concentration, which was consistently over an order of magnitude less than that of the hemiacetals, regardless of the proportions of added reagents. Thus, the observation of hemiacetals at relatively higher abundance than carbonyl HCHO in e-cigarette aerosols is not surprising.

### Incomplete conversion of hemiacetals 1a–d to HCHO under acidic DNPH conditions is observed, leading to underestimation of their levels as HCHO equivalents

When solutions enriched in **1a**–**d** were applied to standard DNPH solutions or DNPH sorbent tubes, 33% of the hemiacetals were not detected (i.e., not converted to HCHO) using the DNPH impinger method, and 46% were not detected via the DNPH sorbent tube method. This suggests that equilibration of the hemiacetals to HCHO was not complete using these methods.

The formation of hydrates as the predominant product between most organic carbonyl compounds and H_2_O is relatively rare. However, methane diol (Fig. 6) is a well-known form of HCHO, and was formed by Balashov simply upon addition of H_2_O to enrich the equilibrium concentration of methane diol. It is also well known that acyclic hemiacetals of organic compounds are relatively unstable. However, as described above, in the case of HCHO, hemiacetals have been shown to predominate over acetals under neutral conditions. These examples highlight the unique properties of HCHO. It is thus not surprising that **1a**–**d** are not cleanly converted to HCHO and PG plus GLY when HCHO reacts with DNPH under the inherently acidic conditions in the cartridge and impinger. In fact, it has been clearly shown that acid catalyzes the conversion of hemiacetals to acetals (Fig. 7)^17^. We examined the conversion of commercial PG/GLY acetal standards to HCHO-DNPH adducts under the standard DNPH impinger and cartridge conditions by HPLC. No HCHO-DNPH adduct was detectable by HPLC as arising from the PG or GLY acetals. Taken together, these observations embody evidence prompting us to conclude that PG and GLY hemiacetal conversion to detectable HCHO and HCHO-DNPH is hindered acid-promoted formation of the corresponding inert acetals.

## Conclusion

In this study, HCHO hemiacetals were detected in e-cigarette aerosols at levels higher than those of carbonyl HCHO. This is in agreement with a prior study of the solution-phase equilibria formed between HCHO, hemiacetals, acetals and methane diol^17^. Although the prior study^17^ establishes precedence for high proportions of **1a**–**d** to HCHO based on fundamental chemical principles, precise levels of products generated in e-cigarette aerosols are subject to intrinsic variability^8^. The potential toxicity of the hemiacetals is not yet conclusively known. Their ability to convert to HCHO after inhalation by humans has also not been investigated. The standard aldehyde measurement protocols that have been used for HCHO detection in e-cigarettes, namely DNPH impingers and DNPH sorbent tubes, underestimated significant levels of HCHO exposure in the form of **1a**–**d**. The nominal levels of **1a**–**d** and HCHO reported herein are reflective of relatively high power levels chosen to facilitate the investigation of analytical methods. However, the values are in range of those determined for HCHO without accounting for **1a**–**d** in recent related investigations comparing e-cigarette devices^10^. During the course of this investigation, a hybrid cold trap/impinger method was used that enabled the detection of both DNPH-reactive and DNPH-unreactive aerosol components to be quantified. In addition to sampling and analysis, many chemical (e.g., acidity) and physical factors can influence the levels and identities of PG and GLY degradation products, including the proportion of hemiacetal to free HCHO. Finally, the finding that there is a significant relative abundance of **1a**–**d** to HCHO in e-cigarette aerosols, in general agreement with another recent report^3^, coupled with the fact that **1a**–**d** are not well-accounted for as HCHO equivalents via widely used analytical methods, necessitates broader investigation. The further study of **1a**–**d** as well as other challenging e-cigarette aerosol toxins, including acrolein and acetaldehyde, will be reported in due course.

## Electronic supplementary material

Supplementary Information
