# Supplementary material for: Formaldehyde Hemiacetal Sampling, Recovery, and Quantification from Electronic Cigarette Aerosols
Source: Sci Rep. 2017 Sep 8;7:11044. doi: 10.1038/s41598-017-11499-0 (PMC5591312; doi:10.1038/s41598-017-11499-0)
Supplement: Supplementary file 1 — Supplementary Information [file 41598_2017_11499_MOESM1_ESM.docx]

**Formaldehyde Hemiacetal Sampling, Recovery, and Quantification from Electronic Cigarette Aerosols**

James C. Salamanca, Ian Munhenzva, Jorge O. Escobedo, R. Paul Jensen, Angela Shaw, Robert Campbell, Wentai Luo, David H. Peyton, and Robert M. Strongin*

**Supporting Information**

**Figure S1.** DNPH-HCHO calibration curve for HPLC analysis.

| Batch J3-FRA-V-β |  |  |  | | Average HCHO (µg) | | Recovery HCHO (%) | |  |
| --- | --- | --- | --- | --- | --- | --- | --- | --- | --- |
|  | Peak Area | HCHO (µg/5mL) | HCHO (µg) | |  | |  | |  |
| **DNPH solution.** | 644123 | 1.02 | **5.10** | |  | |  | |  |
|  | 644593 | 1.02 | **5.11** | |  | |  | |  |
|  | 643311 | 1.02 | **5.10** | |  | |  | |  |
|  |  |  |  | | **5.10** | | **66.79** | |  |
| **Sorbent tubes: T = 0 min** | 519910 | 0.82 | **4.12** | |  | |  | |  |
|  | 521744 | 0.83 | **4.13** | |  | |  | |  |
|  | 512077 | 0.81 | **4.06** | |  | |  | |  |
|  |  |  |  | | **4.10** | | **53.72** | |  |
|  |  |  |  | |  | |  | |  |
| PG-HCHO hemiacetal = 27.0 µg (Equivalent to **7.64** µg HCHO) | | | |  | |  | |  | |

**Table S1.** Percent recovery comparison between DNPH solution and sorbent tubes of enriched PG/GLY-HCHO hemiacetal.

**Table S3.** Data for sampling and analysis for the combined qNMR-DNPH method under vaping conditions.

| **Hybrid System** |  |  |  |  |  |  |
| --- | --- | --- | --- | --- | --- | --- |
| Trial # | Power (W) | E-liq mass collected (mg) | DNPH-HCHO in Impingers (μg) | PG-HA in Cold-traps (μg) | Combined total HCHO (μg) | HCHO μg/mg e-liquid |
| T1 | 10 | 214.39 | 92.57 | 3680.158863 | 3772.728863 | 17.59750391 |
| T2 | 10 | 137.92 | 51.0128 | 2517.38778 | 2568.40058 | 18.622394 |
| T3 | 10 | 192.04 | 505.952 | 2879.965054 | 3385.917054 | 17.63131147 |
|  |  |  |  |  |  |  |
| T1 | 15 | 228.15 | 1122.678755 | 18250.7251 | 19373.40385 | 70.13273059 |
| T2 | 15 | 237.15 | 1314.696889 | 14659.13686 | 15973.83375 | 67.35751108 |
| T3 | 15 | 239.89 | 898.5356869 | 16591.35246 | 17489.88815 | 72.90795009 |

**Table S4.** Data for sampling and analysis for DNPH sorbent tubes.

| **Sorbent Tubes** |  |  |  |  |  |  |
| --- | --- | --- | --- | --- | --- | --- |
| Trial # | Power (W) | Total Mass collected (mg) | DNPH-HCHO in Impingers (μg) | HCHO μg/mg e-liquid |  |  |
| T1 | 10 | 13.59 | 42.84646500 | 4.154159389 |  |  |
| T2 | 10 | 14.71 | 68.31955334 | 4.644459551 |  |  |
| T3 | 10 | 15.67 | 73.09732926 | 4.664964901 |  |  |
|  |  |  |  |  |  |  |
| T1 | 15 | 24.97 | 73.23136562 | 2.932773954 |  |  |
| T2 | 15 | 31.11 | 85.51946854 | 2.748938237 |  |  |
| T3 | 15 | 30.2 | 89.70866993 | 2.718444543 |  |  |
|  |  |  |  |  |  |  |

**Table S5.** Data for sampling and analysis for DNPH solution impingers.

| **DNPH Solution** | |  |  |  |  |  |
| --- | --- | --- | --- | --- | --- | --- |
| Trial # | Power (W) | Total mass collected (mg) | DNPH-HCHO in Impingers (μg) | HCHO μg/mg e-liquid |  |  |
| T1 | 10 | 17.13 | 151.29286868 | 8.832041371 |  |  |
| T2 | 10 | 33.97 | 304.9844439 | 8.978052514 |  |  |
| T3 | 10 | 43.30 | 376.10510887 | 8.686030228 |  |  |
|  |  |  |  |  |  |  |
| T1 | 15 | 14.73 | 196.4943431 | 13.33973816 |  |  |
| T2 | 15 | 48.56 | 706.5608073 | 14.55026374 |  |  |
| T3 | 15 | 53.26 | 646.0018624 | 12.12921259 |  |  |

**Table S6.** Comparison between recovery, repeatability, and limits of detection and quantitation across qNMR and DNPH methods of determining HCHO levels from **1a-d**.

| Method | Recovery  (%) | LOD  (µg/mL) | LOQ  (µg/mL) | RSD  (%, *n* = 3) |
| --- | --- | --- | --- | --- |
| qNMR (1a-d) | 98.90 | 167.7 | 562.2 | 0.40 |
| DNPH impingers (HCHO from 1a-d) | 66.75 | 0.03 | 0.10 | 6.95 |
| DNPH sorbent tubes (HCHO from 1a-d) | 53.66 | 0.03 | 0.10 | 6.95 |
